# A global synthesis and conceptualization of the magnitude and duration of soil carbon losses in response to forest disturbances

**DOI:** 10.1111/geb.13779

**Published:** 2023-10-24

**Authors:** Mathias Mayer, Andri Baltensweiler, Jason James, Andreas Rigling, Frank Hagedorn

**Affiliations:** ^1^ Forest Soils and Biogeochemistry Swiss Federal Institute for Forest, Snow and Landscape Research (WSL) Birmensdorf Switzerland; ^2^ Forest Ecology, Institute of Terrestrial Ecosystems (ITES) ETH Zurich Zurich Switzerland; ^3^ Institute of Forest Ecology, Department of Forest and Soil Sciences University of Natural Resources and Life Sciences (BOKU) Vienna Austria; ^4^ Forest Resources and Management Swiss Federal Institute for Forest, Snow and Landscape Research (WSL) Birmensdorf Switzerland; ^5^ Exponent, Inc. Bellevue Washington USA; ^6^ Forest Dynamics Swiss Federal Institute for Forest, Snow and Landscape Research (WSL) Birmensdorf Switzerland

## Abstract

**Aim:**

Forest disturbances are increasing around the globe due to changes in climate and management, deteriorating forests' carbon sink strength. Estimates of global forest carbon budgets account for losses of plant biomass but often neglect the effects of disturbances on soil organic carbon (SOC). Here, we aimed to quantify and conceptualize SOC losses in response to different disturbance agents on a global scale.

**Location:**

Global.

**Time Period:**

1983–2022.

**Major Taxa Studied:**

Forest soils.

**Methods:**

We conducted a comprehensive global analysis of the effects of harvesting, wildfires, windstorms and insect infestations on forest SOC stocks in the surface organic layer and top mineral soil, synthesizing 927 paired observations from 151 existing field studies worldwide. We further used global mapping to assess potential SOC losses upon disturbance.

**Results:**

We found that both natural and anthropogenic forest disturbances can cause large SOC losses up to 60 Mg ha^−1^. On average, the largest SOC losses were found after wildfires, followed by disturbances from windstorms, harvests and insects. However, initial carbon stock size, rather than disturbance agent, had the strongest influence on the magnitude of SOC losses. SOC losses were greatest in cold‐climate forests (boreal and mountainous regions) with large accumulations of organic matter on or near the soil surface. Negative effects are present for at least four decades post‐disturbance. In contrast, forests with small initial SOC stocks experienced quantitatively lower carbon losses, and their stocks returned to pre‐disturbance levels more quickly.

**Main Conclusions:**

Our results indicate that the more carbon is stored in the forest's organic layers and top mineral soils, the more carbon will be lost after disturbance. Robust estimates of forest carbon budgets must therefore consider disturbance‐induced SOC losses, which strongly depend on site‐specific stocks. Particularly in cold‐climate forests, these disturbance‐related losses may challenge forest management efforts to sequester CO_2_.

## 1 INTRODUCTION

Forests are the largest terrestrial carbon (C) pool on Earth, with about 30%–70% of organic C stored below ground, making forest soils an important terrestrial sink for atmospheric CO_2_ (Pan et al., 2011). Theoretical concepts suggest a slow build‐up of soil organic C (SOC) stocks with stand growth and development, usually reaching equilibrium in a late‐successional forest state (Chapin III et al., 2002). However, these concepts also assume that forests can abruptly lose SOC when they are disturbed by harvesting or natural agents, such as fires, windstorms, or insect infestations. The SOC loss induced by forest disturbance through harvesting or natural agents is related to reduced input of fresh plant litter to the soil following tree removal or death and to continuing C fluxes out of the soil, e.g. through microbial decomposition, burning, or erosion (Gerber et al., 2002; Holloway et al., 2020; Mayer, Sandén, et al., 2017). Moreover, physical disturbance of soil structure may increase the availability of protected organic matter (Cambi et al., 2015; Kramer et al., 2004), and a more favourable soil climate through the loss of a tree canopy may further accelerate microbial C mineralization (Mayer, Sandén, et al., 2017). Disturbance‐induced SOC losses are suggested to be quantitatively important, with released CO_2_ exacerbating climate warming (Kurz et al., 2008).

Natural disturbances have increased in frequency, severity and extent in recent decades, and this trend is predicted to continue, with human land use and climate change regarded as the main drivers (Patacca et al., 2022; Seidl et al., 2017; Senf & Seidl, 2021). In addition, forestry has been estimated to account for 26% of the world's forest cover change between 2001 and 2015, making timber harvesting a globally important driver of forest disturbance (Curtis et al., 2018). While forest carbon budgets at the continental and global scale account for C losses through tree biomass death or removal following disturbances, they most often do not consider the effects of disturbances on SOC (Roebroek et al., 2023; Wang et al., 2023). One of the reasons might be that, despite decades of scientific attention (Covington, 1981; Li et al., 2021; Nave et al., 2011; Yanai et al., 2003; Zhang et al., 2015), there is no consensus on the magnitude and duration of SOC losses in response to different disturbance agents. For example, earlier meta‐analyses of the response of SOC to harvesting suggest no overall effects on C stocks (Johnson & Curtis, 2001) or that C losses occur only in the organic layer (Nave et al., 2010). The IPCC (2019) inventory guidelines therefore assume that forest management does not affect mineral SOC stocks. However, more recent meta‐analyses on the impacts of harvesting show that C losses can also occur in the mineral soil and that SOC stocks can be reduced for decades upon harvest (James et al., 2021; James & Harrison, 2016). Similarly, global syntheses of the effects of wildfire (Li et al., 2021; Nave et al., 2011), insect infestations (Kristensen et al., 2019; Zhang et al., 2015) and larger scale studies on windthrow (Mayer et al., 2023) show inconclusive results, ranging from large and long‐lasting C losses to even increased SOC stocks after disturbance. Finally, meta‐analyses assessing the impact of both human and natural disturbance on forest SOC stocks are rare and confined to specific regions (Nave, DeLyser, Domke, Holub, Janowiak, Kittler, et al., 2022; Nave, DeLyser, Domke, Holub, Janowiak, Ontl, et al., 2022). These shortcomings limit generalization and the validation of Earth system models and larger scale estimates of forest C sink capacities (Cook‐Patton et al., 2020; Harris et al., 2021; Lindroth et al., 2009; Pugh et al., 2019; Roebroek et al., 2023). Thus, a globally comprehensive and spatially explicit assessment of the sensitivity of SOC stocks to major forest disturbance agents and an analysis of the factors underlying the magnitude and duration of SOC losses in response to disturbance is urgently needed. This knowledge is a prerequisite for identifying forest ecosystems that are most vulnerable to large and long‐lasting SOC losses after disturbances.

To investigate the impact of anthropogenic and natural disturbance on forest SOC on a global scale, we synthesized data from 151 published field studies on the effects of harvesting, wildfires, windstorms and insect infestations on SOC stocks. The studies covered the world's major forest biomes, with a strong focus on boreal and temperate forests in the northern hemisphere (Table S1). Based on data availability across disturbance studies, we concentrated on the organic layer and top mineral soil. In total, we analysed 542 observations from the organic layer and 385 from the top mineral soil. In contrast to earlier meta‐analyses, we focused only on observations of C stocks (expressed in Mg ha^−1^), excluding observations of C concentrations (g g^−1^). We used these data to quantify absolute SOC stock changes, estimated as the difference in SOC stock between paired undisturbed reference stands (control) and disturbed stands (treatment). We aimed to address four specific research questions:

1. What are the quantitative effects of harvesting, wildfires, storms and insect infestations on SOC stocks and do these agents differ in their influence?
2. What factors drive the magnitude and duration of SOC losses in response to disturbances?
3. Which forest ecosystem types lose the most SOC upon disturbance?
4. How long does it take for SOC stocks to return to pre‐disturbance levels?

## 2 MATERIALS AND METHODS

### 2.1 Data set

We collated the references of recent peer‐reviewed meta‐analyses on the effects of forest harvesting (Hume et al., 2018; James et al., 2021; James & Harrison, 2016; Nave, DeLyser, Domke, Holub, Janowiak, Kittler, et al., 2022; Nave, DeLyser, Domke, Holub, Janowiak, Ontl, et al., 2022), wildfire (Li et al., 2021; Nave, DeLyser, Domke, Holub, Janowiak, Kittler, et al., 2022; Xu et al., 2022), insect infestations (Kristensen et al., 2019; Zhang et al., 2015) and windstorms (Mayer et al., 2023), and filtered them for studies reporting SOC stocks (Mg ha^−1^). Studies reporting SOC concentrations (g g^−1^) together with soil bulk density (g cm^−3^) and layer thickness (cm) were also extracted, which were then used to calculate SOC stocks. Studies reporting only SOC concentrations were not included as they do not allow quantification of C stock changes and may even be misleading when original organic layer masses but not concentrations change after disturbances.

We focused on upper soil horizons, where soil depths were separated into an organic layer and top mineral soil (0–10 cm). If SOC stocks for multiple organic layers (e.g. O_L_, O_F_) or mineral soil depths (e.g. 0–5, 5–10 cm) were available, SOC stocks were summed up for the soil layers. Studies reporting top mineral SOC stocks for smaller (e.g. 0–7 cm) or larger (e.g. 0–12 cm) sampling depths were also included in our analysis. There was less data available and large variability in soil sampling depth for deeper horizons (e.g. 10–20 cm, 10–50 cm) across studies, impeding a valid comparison of absolute C losses. Consequently, we did not include these horizons in our analysis. To be included in our analysis, studies had to report both SOC stocks for an undisturbed reference stand (control) as well as for a disturbed stand (treatment). For forest chronosequence studies, SOC stocks of the oldest stands were used as the control stands. In cases of multiple disturbances (e.g. wildfire after windthrow), the agent that occurred first was selected.

We complemented the data set from previous meta‐analyses with additional studies on the effects of wildfires, insect infestations and windstorms on SOC stocks found through a literature search using the Scopus database and Google scholar. The search terms were ‘wind disturbance’, ‘windthrow’, ‘windbreak’, ‘storm’, ‘blowdown’, ‘wildfire’, ‘forest fire’, ‘bark beetle’, ‘insect infestation’, ‘insect outbreak’ and ‘soil carbon’. If results were shown in figures only, SOC stock data were extracted using open‐source graph digitizer software (plotdigitizer.com).

In total, we synthesized data from 151 field studies on the effects of forest harvesting (*n* = 89), wildfire (*n* = 48), insect infestations (*n* = 18) and windstorms (*n* = 13). The studies covered temperate forests (*n* = 113), boreal forests (*n* = 27) and tropical and subtropical forests (*n* = 11). Most of the studies were conducted in North America, followed by Europe, Asia, South America, Australia and Africa (Table S1). In total, we used 542 and 385 observations from the organic layer and mineral soil, respectively. For each study, we extracted information on the disturbance agent (harvesting, insects, wind and wildfire), biome (boreal, temperate and tropical/subtropical), forest type (coniferous, broadleaf and mixed), time since disturbance (in years) and mean annual temperature (MAT) and precipitation (MAP).

### 2.2 Data analysis

Prior to analysis, the SOC stocks from individual studies were all converted to the unit Mg ha^−1^. Absolute SOC stock changes in response to forest disturbance agents were subsequently calculated as:

(1) $$\mathrm{SOC}\;\text{stock change}={\bar{X}}_{D}-{\bar{X}}_{C}$$

where ${\bar{X}}_{D}$ and ${\bar{X}}_{C}$ are the mean values of SOC stocks of disturbed areas and control stands, respectively, of each study site and soil layer (i.e. organic layer/mineral soil). If a study included multiple sites with different disturbance agents (e.g. wildfires and windstorms), separate values were calculated. Estimates of variance (e.g. standard error) and sample size were not available in many publications. The use of classic meta‐analysis approaches was therefore not possible (Hedges et al., 1999). Instead, we used linear mixed effects (LME) modelling for data analysis (Pinheiro & Bates, 2000). Individual studies were considered as random effects in each model.

First, we used non‐intercept models to test whether SOC stock changes grouped after disturbance agent, biome and forest type differed significantly from zero. We conducted similar tests for three SOC stock levels (small, medium and large) and four classes of time since disturbance. For this, we grouped the observations according to their initial SOC stock (i.e. control stand stock) using their 33rd and 66th percentile ranks and according to the time since disturbance (<10, 10–25, 25–40 and >40 years); a lack of data did not allow for a further temporal separation into the individual disturbance agents.

We then used LME modelling to test the significance and explanatory power of single and multiple predictors on SOC stock change. The considered predictors were disturbance agent, biome, forest type, MAT, MAP, time since disturbance and control stand SOC stock. We used marginal pseudo‐*R* ^2^ values for model assessment (Nakagawa & Schielzeth, 2013).

### 2.3 Mapping of soil organic carbon loss

To visualize SOC loss on a global scale, we used the relationship between SOC stock change and control stand SOC stocks in the organic layer and top mineral soil. A non‐linear power function was fitted to the data. We estimated the 95% confidence interval of the predictions using a bootstrapping approach (Loy & Korobova, 2021). We did not test the robustness of the relationship due to the lack of variance estimates and sample sizes for many studies (Hong et al., 2020; Slessarev et al., 2023).

We applied the SOC response functions to a global map of SOC stocks (Hengl et al., 2017), which we masked with a global forest map (Sayre et al., 2020). The global map of SOC stocks does not distinguish between organic and mineral soil layers. Therefore, we applied the SOC response functions for organic and mineral soil layers to available C stocks in 0–5 cm and 5–15 cm depths, respectively. We considered a prediction to be a significant SOC loss only when the bootstrapped confidence interval was below zero. Moreover, we set predicted SOC losses outside the data range of the models to the maximum predicted values within their prediction range. We then calculated and mapped total SOC losses (summed across the two soil depth ranges).

We conducted all statistical analyses in R (R Core Team, 2021) using the packages ‘nlme’ (Pinheiro et al., 2017), ‘lme4’ (Bates et al., 2009), ‘MuMIn’ (Barton, 2009) and ‘lmeresampler’ (Loy & Korobova, 2021). The level of significance for all statistical analyses was set at *p* < 0.05.

## 3 RESULTS AND DISCUSSION

**FIGURE 1:**
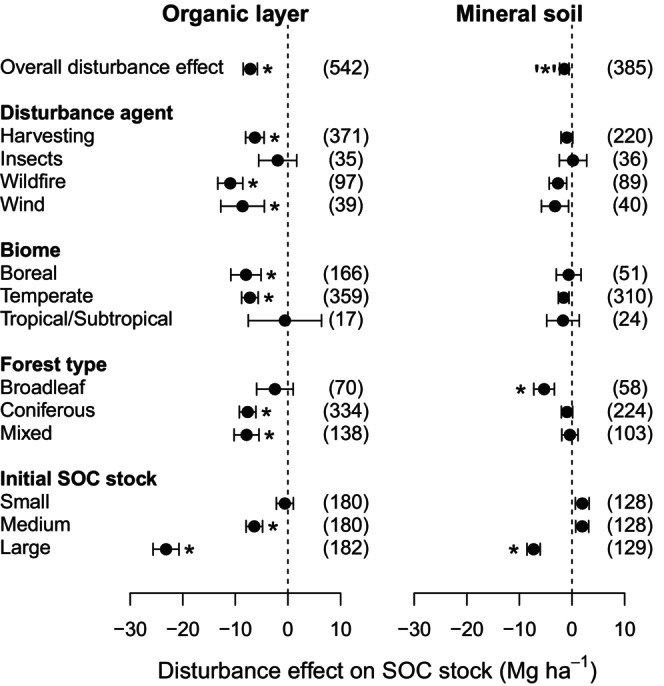
Effect of forest disturbance on soil organic carbon (SOC) stock in the organic layer and top mineral soil across different factors. Means and standard errors based on linear mixed effects models are displayed. The number of observations is given in parentheses. Significant differences from zero are indicated with asterisks (*p < 0.05; ‘*’*p* < 0.1). Initial SOC stocks are calculated as stocks of undisturbed stands (control) grouped as intervals of equal sample size. The ranges of these SOC stock groups are given in Figure 4.

**FIGURE 2:**
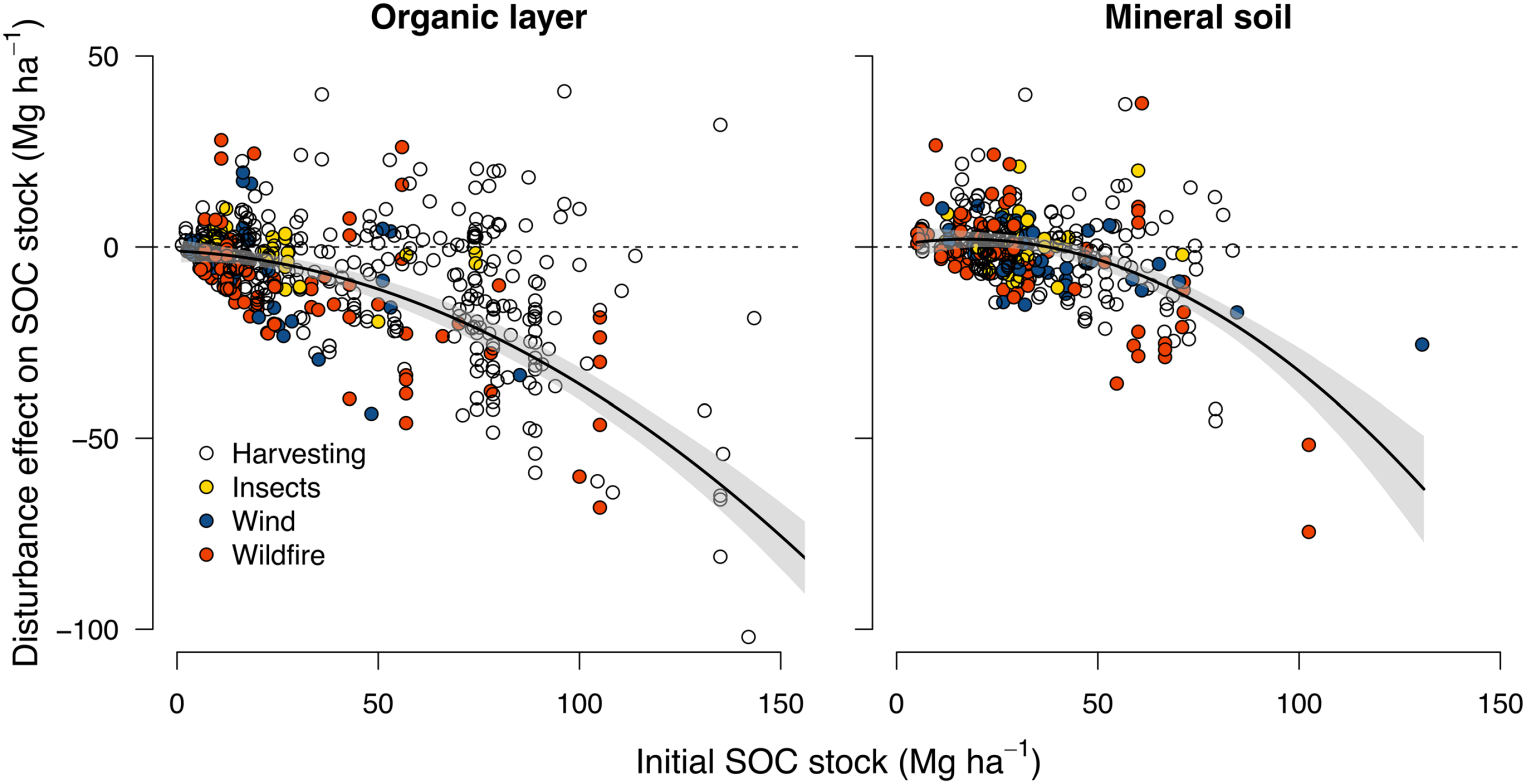
The effect of forest disturbances on soil organic carbon (SOC) losses depends on the initial SOC stock. The relationships were fitted with a non‐linear mixed effects model (organic layer *n* = 542, *R* ^2^ = 0.49; mineral soil *n* = 385, *R* ^2^ = 0.24). The shaded area represents the bootstrapped 95% confidence interval. Different disturbance agents are shown in different colours.

Our analysis showed that forest disturbance can cause considerable SOC losses, with greater impacts occurring in the organic layer than in mineral soil (Figures 1 and 2). On average, forest disturbance decreased the SOC stock in the organic layer by 7.2 ± 1.3 Mg ha^−1^ or 25.7% (Figure 1), suggesting large C losses after stand damage (*p* < 0.001). In mineral soil, in contrast, disturbance decreased SOC stocks only marginally, by 1.5 ± 0.9 Mg ha^−1^ or 4.1% (*p* < 0.1). Carbon losses from the organic layer were greatest after wildfire (11.0 ± 2.4 Mg ha^−1^; −41.1%), followed by damage from windstorms (8.6 ± 4.1 Mg ha^−1^; −25.0%), harvesting (6.3 ± 1.8 Mg ha^−1^, −22.7%) and insect infestations (2.0 ± 3.6 Mg ha^−1^, −7.7%). Although the differences between disturbance agents were not statistically significant (*p* = 0.146, Figure S1), the order of their effect sizes was consistent with meta‐analyses from North America showing stronger effects of fire than harvesting on SOC stocks (Nave, DeLyser, Domke, Holub, Janowiak, Kittler, et al., 2022; Nave, DeLyser, Domke, Holub, Janowiak, Ontl, et al., 2022). It seems plausible that the thermal impact of burning on SOC is more severe than other disturbance effects, such as increased decomposition and erosion (Abney et al., 2017; Gerber et al., 2002; Mayer, Matthews, et al., 2017). Following windthrow and the associated uprooting of trees, C losses from soils may also result from physical soil disturbance, loosening, fracturing and mixing of soil horizons and aggregates (Kooch et al., 2014; Šamonil et al., 2010). In contrast, SOC losses were not statistically significant following insect infestations. Inputs from frass deposits (e.g. litter, faeces) may offset C losses related to insects to a certain degree (Le Mellec et al., 2009; Yang & Gratton, 2014), keeping total SOC stocks closer to pre‐disturbance levels. Soil organic C losses were weakly related to time since disturbance, forest type, MAT and MAP (Figures 1 and S1). Overall, the explanatory power of disturbance agents, climate variables and forest type for predicting SOC losses was low (*R* ^2^_marginal_ = 0.01–0.12, Figure S1).

By far the best predictor of SOC change in the organic layer and in mineral soil was the initial SOC stock prior disturbance (measured as the SOC stock of control; Figure S1), with larger SOC losses with increasing size of the initial stock. Fitting a non‐linear function to the relationship explained 49% and 24% of the variation in SOC loss from the organic layer and mineral soil, respectively. Adding other predictors or interaction terms improved the predictions only marginally (Figure S1). A relationship between SOC loss and initial SOC stock was also present when the disturbance agents were analysed separately and when C stock changes were analysed on a relative basis, indicating that the fraction of SOC lost upon disturbance increased with increasing C stocks (Figures S2 and S3). Only in the case of insect damage was the relationship less evident, but this could be due to the lack of insect‐damaged sites with large SOC stocks (Figure S2).

**FIGURE 3:**
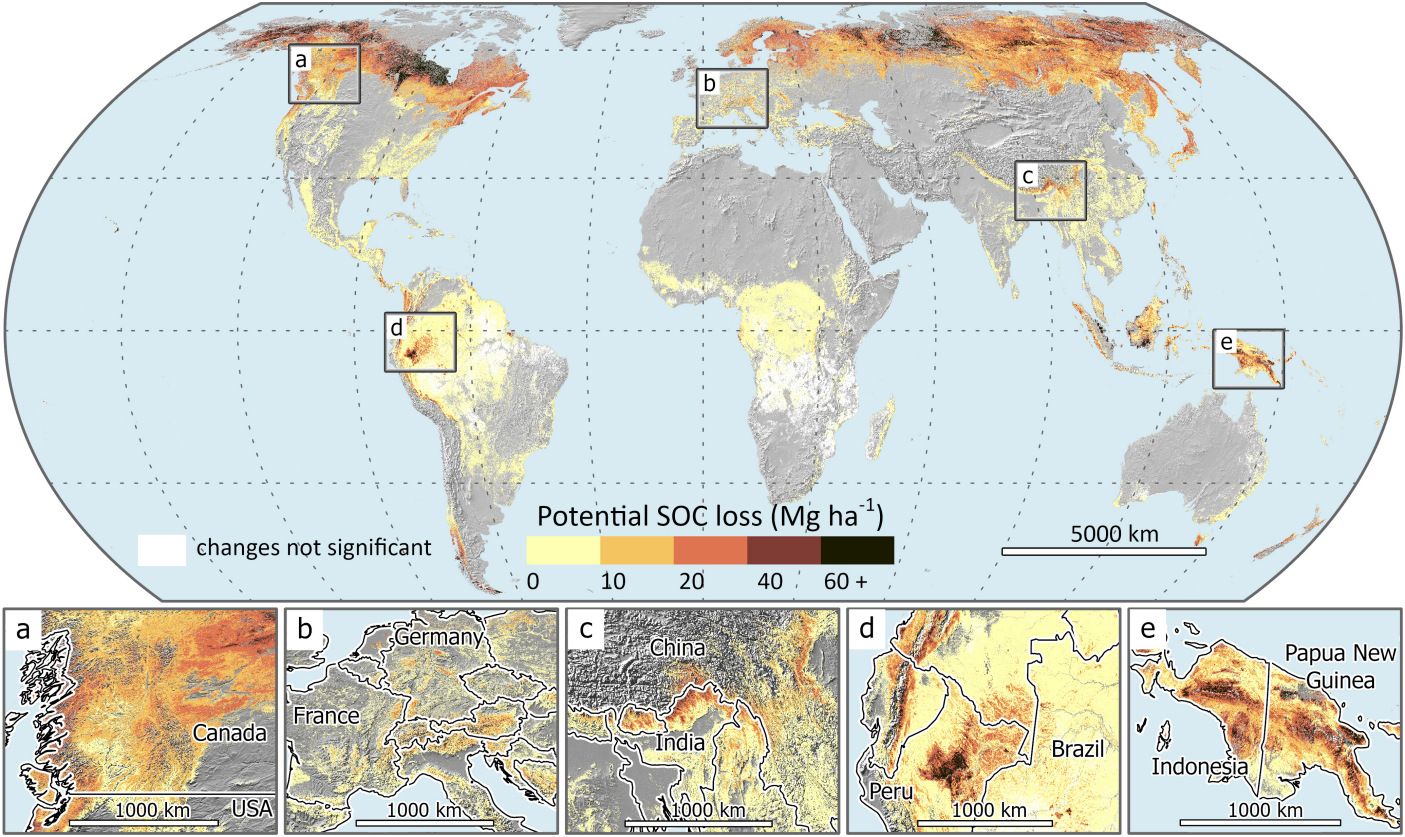
Global map of potential soil organic carbon (SOC) loss upon forest disturbance. The map was generated by applying SOC loss response models (Figure 2) to a global database of SOC stocks masked with a global forest map. Soil organic C losses are calculated for 0–5 cm (representing the organic layer) and 5–15 cm (representing top mineral soil) soil depths and summed for the total losses displayed here. The lower panels show detailed areas of the global map with a high potential for SOC loss upon forest disturbance.

The dominant role of the initial C stock in determining SOC loss implies that C dynamics in response to disturbance depend more strongly on site‐specific SOC quantities than on the disturbance agent or other environmental variables. This finding is in good agreement with studies on climate warming, CO_2_ fertilization and afforestation, which show that the magnitude of SOC changes is congruent with the size of the SOC stock (Hong et al., 2020; Prietzel et al., 2016; Terrer et al., 2021). Thus, forests characterized by a thick organic layer and C‐rich mineral soil appear most vulnerable to C loss in the face of environmental change, including natural disturbances. The results also suggest that in forests with inherently large SOC stocks, harvesting operations can induce large C losses. In contrast, in forests with small soil C stocks, natural disturbance and harvesting lead to smaller C releases from the soil. To identify global hotspots of potential SOC loss upon forest disturbance, we applied the SOC response functions from our models (Figure 2) to a global database of SOC stocks (Hengl et al., 2017). Masking the resulting map with a global forest map (Sayre et al., 2020) showed that boreal forests were the most vulnerable to SOC loss of all forest biomes worldwide (Figure 3) as they store the largest amount of soil C in their uppermost soil layers (Pan et al., 2011). Most of the boreal region would experience tremendous C losses from the organic layer and top mineral soil upon forest disturbance, ranging between 20 and 60 Mg C ha^−1^. Considering that boreal forests store an average of 50 to 70 Mg C ha^−1^ in their vegetation (Bradshaw & Warkentin, 2015), these losses correspond to about 30% to 85% of the aboveground biomass stocks. In contrast, in forests with smaller SOC stocks, such as those in many temperate, tropical and subtropical regions, SOC losses would be comparatively smaller or undetectable in our analysis (i.e. within the bootstrapped 95% confidence interval). However, even in these biomes, there were sites with large accumulations of SOC that could release a substantial amount of SOC after disturbance. These include mountain forests in the North American Cascades (Figure 3a), the European Alps (Figure 3b), the Himalayan region (Figure 3c) and forests in the north‐eastern Amazon (Figure 3d). Tropical forests with peaty soils in southeast Asia (Figure 3e) were also characterized by large and thus highly vulnerable SOC stocks (Yuwati et al., 2021).

**FIGURE 4:**
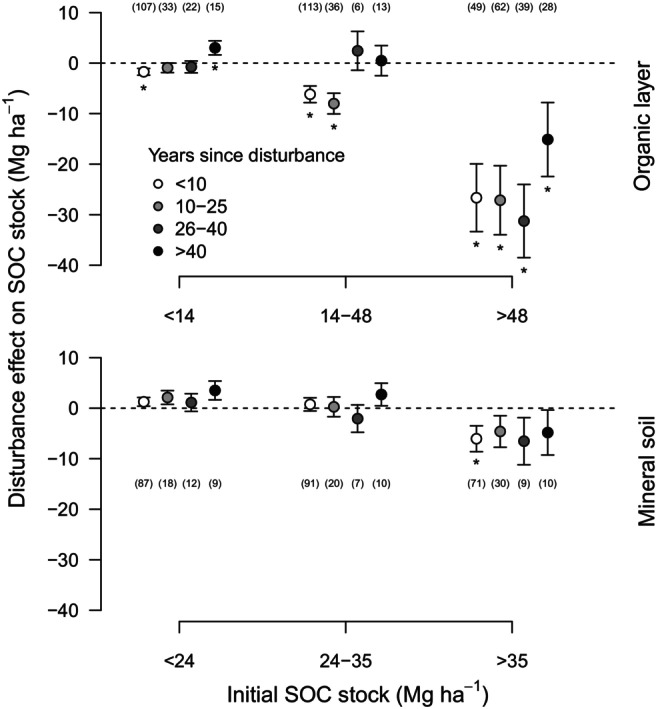
The effect of forest disturbances on soil organic carbon (SOC) stock depends on the initial stock and differs with time since disturbance. Analyses of SOC stocks in the organic layer (top) and mineral soil (bottom) are based on linear mixed effects models, with means and standard errors displayed. The number of observations for each time class is given in brackets. Significant (*p* < 0.05) differences from zero are indicated with asterisks. Initial SOC stocks are calculated as control (undisturbed) stand stocks grouped as intervals of equal sample size (see Figure 1).

The size of initial SOC stocks also determined the duration of SOC change following disturbance (Figure 4). In forests storing small amounts of C in organic layers, C stocks were reduced only during the first 10 years post‐disturbance, while thereafter C stocks were similar to or larger than prior to disturbance. In forests storing larger amounts of SOC, in contrast, the negative effects of disturbance on SOC stocks were present even 40 years post‐disturbance. This suggests that the reduced C inputs to the soil cannot keep pace with the C losses after disturbance, resulting in large and long‐lasting C losses. The reduction in SOC stock can persist for up to a century, as observed for windthrown forests in Alaska (Kramer et al., 2004), but studies involving longer post‐disturbance times are rare.

**FIGURE 5:**
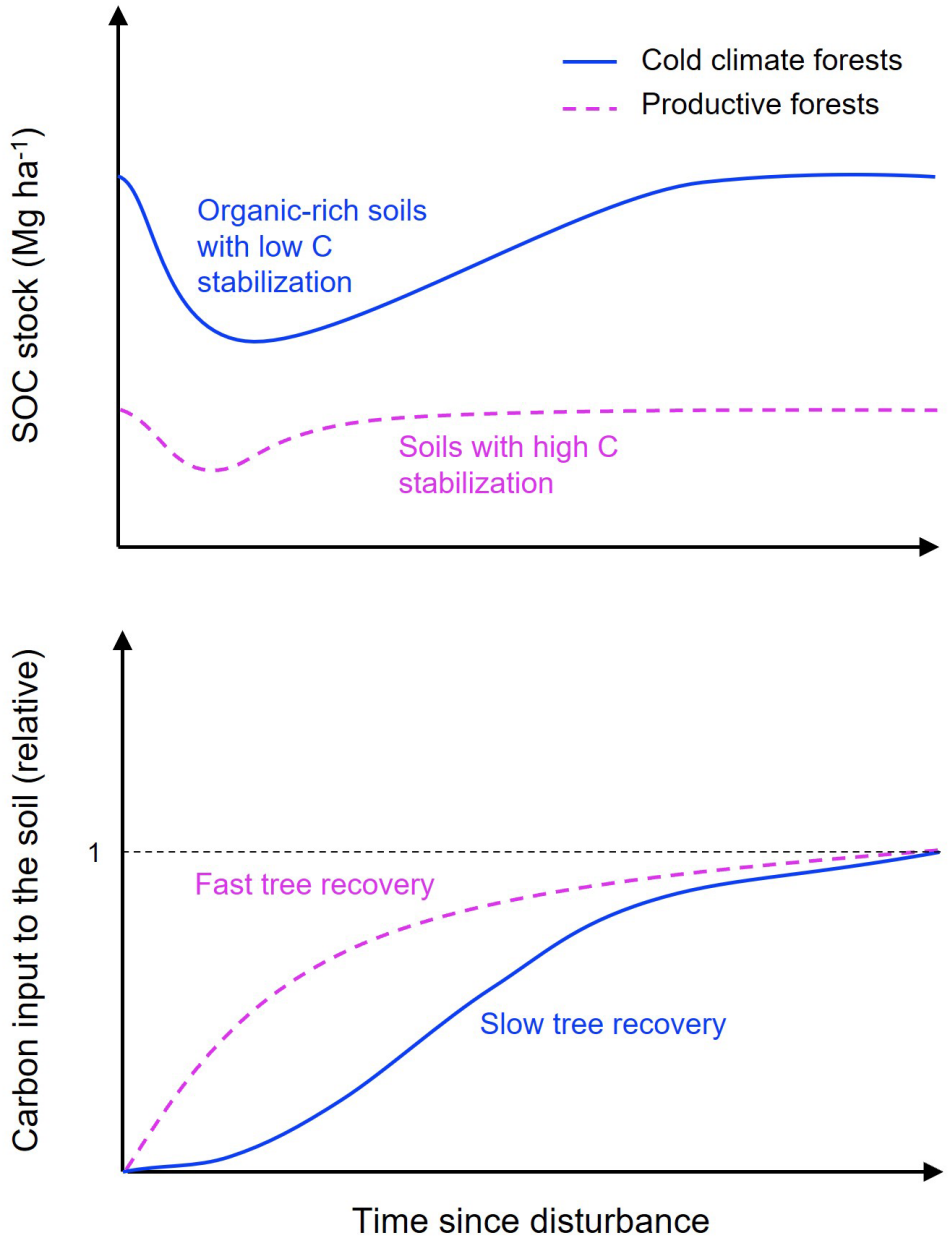
Conceptual summary of changes in soil organic carbon (SOC) stocks in response to forest disturbance and how SOC losses relate to initial stock size, carbon (C) stabilization and forest regrowth dynamics following stand damage.

We propose two explanations for the higher sensitivity of boreal and mountain forests to large and long‐lasting SOC loss after disturbance relative to forests in other regions (Figure 5). First, these forest types are characterized by the highest stocks of ‘labile’ carbon in readily decomposable organic matter. The cold conditions in boreal and mountain forests, in conjunction with the adverse chemical composition of litter from coniferous trees and ericaceous shrubs, typically dominating the vegetation in these ecosystems, hamper the decomposition of annual plant‐derived C inputs. In addition, faunal activity and abundance, especially of earthworms that incorporate litter into the mineral soil, are usually low in these regions (Phillips et al., 2019). In the long term, this leads to the accumulation of thick organic layers and the formation of C‐rich top mineral soils (Crowther et al., 2019; Hagedorn et al., 2019; Lugato et al., 2021). Adsorption onto mineral surfaces and inclusion into aggregates are the primary mechanisms protecting organic matter against microbial decomposition (Lehmann & Kleber, 2015; Prietzel et al., 2020). Therefore, C stability is particularly low in the organic layer with small amounts of reactive minerals (Prietzel et al., 2020). Moreover, in mineral soils, the fraction of mineral‐associated stable C decreases with increasing C content, while the fraction of particulate, labile soil C increases (Lugato et al., 2021). Many boreal and mountain forests also grow on relatively young soils that have developed only since the beginning of the last interglacial period and thus have experienced relatively little secondary clay or iron/aluminium oxide formation. Accordingly, larger C stocks in both organic layer and mineral soils, as in boreal and mountain forests, are more susceptible to disturbance‐induced C losses through microbial decomposition, but likely also through burning and erosion (Figure 5). In comparison, the soils of forests in many temperate and tropical regions have generally thin organic layers but higher contents of mineral‐associated organic C, which promotes soil C stabilization (Georgiou et al., 2022; Lugato et al., 2021; Pan et al., 2011). The warmer soil temperatures and soil moisture following forest disturbance by the tree canopy loss and decrease in transpiration further accelerate microbial processing and associated SOC losses after disturbance (Mayer, Sandén, et al., 2017).

A second explanation for large and long‐lasting disturbance‐induced SOC losses in high‐latitude boreal and high‐elevation mountain forests is hampered forest regeneration (Figure 5) under cold conditions and short growing seasons (Keeling & Phillips, 2007; Kramer et al., 2014). The low net primary productivity and slow tree establishment constrain litter production from forest regrowth, and therefore, the associated replenishment of SOC is slower in these cooler ecosystems. Moreover, the phase of soil exposure to irradiation and precipitation of disturbed land is prolonged with slowed forest regrowth, which in turn can promote post‐disturbance decomposition and erosion (Gerber et al., 2002; Mayer, Matthews, et al., 2017). In comparison, in more productive ecosystems, such as many temperate and tropical forests, plant C derived from forest regrowth accumulates more rapidly (Cook‐Patton et al., 2020), with new C inputs to the soil offsetting C losses more quickly (Figure 5). This might also explain why the SOC stocks of tropical and subtropical forests were not significantly affected by disturbance in our analysis (Figure 1).

Climatic changes and intensifying disturbance regimes (i.e. more severe and more frequent) are expected to change forest's structure and composition, forcing forests globally towards shorter statured and/or younger stands (McDowell et al., 2020; Seidl & Turner, 2022). It is thus unclear whether the C stocks of severely affected forest soils will be able to fully recover after disturbances under future forest dynamics. If they do not regain their initial stock size, and if forest biomass growth cannot compensate for the lost SOC, this amount of C is ‘irrecoverably’ lost to the atmosphere, resulting in positive climate feedback (Goldstein et al., 2020).

We emphasize that our global analysis has several limitations. First, our analysis does not account for disturbance severity and extent (i.e. area of damage), disturbance frequency (i.e. reoccurrence), or post‐disturbance management (e.g. salvage logging or tree planting), as this information was often not provided in the studies. Particularly, fire severity has been shown to affect the magnitude of SOC loss (Li et al., 2021). In addition, our analysis does not differentiate between harvesting techniques such as whole‐tree and stem‐only harvesting or clearcutting versus partial cutting. Interestingly, lower intensity harvesting techniques have been reported to potentially result in similar or even more negative effects on total SOC stocks (James & Harrison, 2016). The highest SOC losses observed in our analysis, for example, occurred after selective harvesting in mountain forests in the Bavarian Alps (Christophel et al., 2013). However, these forest soil had an extraordinary thick organic layer and thus contained large amounts of carbon in particulate labile form (Christophel et al., 2013), which may superimpose harvesting intensity effects. Our analysis is further limited to the uppermost soil layers due to limited data availability. However, deeper soil (>30 cm depth) has also been found to be sensitive to intense harvesting disturbances in some of the few studies that examined deeper soils (James & Harrison, 2016). We also stress that tropical and subtropical forests are underrepresented in our analysis due to a lack of available studies (only 7% of all studies). Therefore, the results for these biomes must be considered with caution, and variability might be underestimated. Especially in tropical forests, intensive logging combined with inadequate restoration or land‐use change can lead to severe and long‐lasting site degradation, including soil erosion and long‐lasting SOC loss (Don et al., 2011; Mayer et al., 2020). For example, the conversion of primary to secondary forests has been estimated to result in SOC losses of about 10 Mg ha^−1^ and losses associated with conversion to cropland are even greater (Don et al., 2011). Studies on such land‐use change were not considered in our analysis. We would also like to emphasize that although lower SOC losses may have a smaller impact on atmospheric CO_2_ levels, this does not mean that disturbance is of less concern for ecosystems. For example, in unproductive forests with low nutrient availability, even small disturbance‐induced losses of organically bound nutrients may have negative consequences for their productivity (Pellegrini & Jackson, 2020). Finally, our SOC response functions (Figure 2) were not tested for robustness and were not statistically corrected as previously suggested (Slessarev et al., 2023), because required parameters (i.e. variance, number of replicates) were not available for many studies. We are aware that statistical artefacts due to random spatial variation can potentially bias a relationship between SOC stock size and C changes (Slessarev et al., 2023). However, the increase in SOC losses with increasing SOC stocks existed for each disturbance type and the entire data set, strongly suggesting that the patterns are not driven by a few studies with great leverage. Moreover, our results are in good agreement with other studies showing SOC losses in response to environmental change relate to initial stock size (Hong et al., 2020; Prietzel et al., 2016; Terrer et al., 2021), giving confidence that this pattern also holds true for the effect of disturbance.

Despite these limitations, our findings provide a first conceptual and applicable framework for explaining global SOC changes in response to forest disturbance. The framework is based solely on SOC quantities, the best predictor for SOC loss in response to disturbance, and potentially enables the inclusion of disturbance‐induced SOC losses in spatially explicit Earth System Models and other upscaling approaches. For example, recent studies simulating forest C sink capacities at continental and global scales have either excluded disturbance‐induced SOC losses (Wang et al., 2021; Wu et al., 2023) or assumed, congruent with the IPCC guidelines (IPCC, 2019), that forest management does not affect C stocks in mineral soils (Harris et al., 2021). Our results indicate that forest C sink strengths may be overestimated in boreal and mountain regions if disturbance‐related changes in SOC are not accounted for appropriately. However, in good agreement with our estimates for North American boreal forests, a recent simulation study accounting for wildfire effects on SOC estimated belowground C losses of up to 60 Mg ha^−1^ for this region (Zhao et al., 2021).

In conclusion, our global analysis provides evidence that SOC loss in response to different disturbance agents can be substantial but varies strongly among forest ecosystems. We find that the amount of C stored in the soils is the best predictor of the magnitude of SOC losses. Forests storing large amounts of SOC in organic layers and top mineral soils, such as boreal and mountain forests, represent potential global hotspots for large and long‐lasting C loss after natural disturbance and harvesting. In contrast, forests containing smaller amounts of C in the uppermost soil layers have less C to lose, and their SOC stocks recover faster (Figure 5). Mechanistically, our findings are possibly related to the degree of SOC stabilization as well as the rate of forest regrowth and associated SOC replenishment, which are lower in cold boreal and mountain forests and higher in productive forests. As the frequency and severity of natural forest disturbances are predicted to increase in the future, it is likely that forests with C‐rich topsoils will be at risk of losing large amounts of C, thereby weakening their C sink strength. Thereby, disturbance‐induced SOC losses may also challenge forest management efforts to sequester atmospheric CO_2_. Our findings further indicate that harvesting should be avoided or conducted with caution in forests with inherently high topsoil C stocks. However, in productive forests with lower SOC stocks in the organic layer and top mineral soil, the effect of harvesting on SOC storage appears to be smaller but may still negatively affect other soil functions. We suggest that robust predictions of forest C budgets must account for disturbance‐induced SOC loss, which strongly depends on site‐specific SOC stocks. Finally, we highlight the need for additional long‐term field measurements in regions where current data availability is low, such as forests in tropical and subtropical regions.

## AUTHOR CONTRIBUTIONS

Mathias Mayer, Andreas Rigling and Frank Hagedorn designed the study. Mathias Mayer and Jason James prepared the data base. Mathias Mayer analysed the data. Andri Baltensweiler conducted global upscaling and mapping and contributed to the analysis. All authors contributed to the data interpretation and the writing of the paper.

## CONFLICT OF INTEREST STATEMENT

None to declare.

## Supporting information


**Table S1.** Number of studies per continent considered in the analysis.
**Figure S1**. Explanatory power of models predicting soil organic carbon (SOC) loss from the organic layer and from mineral soil after forest disturbance.
**Figure S2**. Soil organic carbon (SOC) losses in relation to initial SOC stock, separated by disturbance agent and soil layer.
**Figure S3**. Forest disturbance effect on relative changes in soil organic carbon (SOC) stock depends on the initial SOC stock.

**Table S2.** Soil organic carbon stocks in control and disturbed forest sites used in the analysis.

## Data Availability

Data used in this study are available in the Supplementary information (Table S2).

## References

[geb13779-bib-0001] Abney, R. B. , Sanderman, J. , Johnson, D. , Fogel, M. L. , & Berhe, A. A. (2017). Post‐wildfire erosion in mountainous terrain leads to rapid and major redistribution of soil organic carbon. Frontiers in Earth Science, 5, 99.

[geb13779-bib-0002] Barton, K. (2009). MuMIn: Multi‐model inference . http://r‐forge.r‐project.org/projects/mumin/

[geb13779-bib-0003] Bates, D. , Maechler, M. , Bolker, B. , Walker, S. , Christensen, R. H. B. , Singmann, H. , Dai, B. , Scheipl, F. , & Grothendieck, G. (2009). Package ‘lme4’ . http://lme4.r‐forge.r‐project.org

[geb13779-bib-0004] Bradshaw, C. J. A. , & Warkentin, I. G. (2015). Global estimates of boreal forest carbon stocks and flux. Global and Planetary Change, 128, 24–30.

[geb13779-bib-0005] Cambi, M. , Certini, G. , Neri, F. , & Marchi, E. (2015). The impact of heavy traffic on forest soils: A review. Forest Ecology and Management, 338, 124–138.

[geb13779-bib-0006] Chapin, F. S., III , Matson, P. A. , & Mooney, H. A. (2002). Principles of terrestrial ecosystem ecology. Springer.

[geb13779-bib-0007] Christophel, D. , Spengler, S. , Schmidt, B. , Ewald, J. , & Prietzel, J. (2013). Customary selective harvesting has considerably decreased organic carbon and nitrogen stocks in forest soils of the Bavarian limestone Alps. Forest Ecology and Management, 305, 167–176.

[geb13779-bib-0008] Cook‐Patton, S. C. , Leavitt, S. M. , Gibbs, D. , Harris, N. L. , Lister, K. , Anderson‐Teixeira, K. J. , Briggs, R. D. , Chazdon, R. L. , Crowther, T. W. , & Ellis, P. W. (2020). Mapping carbon accumulation potential from global natural forest regrowth. Nature, 585(7826), 545–550. 32968258 10.1038/s41586-020-2686-x

[geb13779-bib-0009] Covington, W. W. (1981). Changes in forest floor organic matter and nutrient content following clear cutting in northern hardwoods. Ecology, 62, 41–48.

[geb13779-bib-0010] Crowther, T. W. , van den Hoogen, J. , Wan, J. , Mayes, M. A. , Keiser, A. D. , Mo, L. , Averill, C. , & Maynard, D. S. (2019). The global soil community and its influence on biogeochemistry. Science, 365(6455), 1–10. 10.1126/science.aav0550 31439761

[geb13779-bib-0011] Curtis, P. G. , Slay, C. M. , Harris, N. L. , Tyukavina, A. , & Hansen, M. C. (2018). Classifying drivers of global forest loss. Science, 361(6407), 1108–1111. 30213911 10.1126/science.aau3445

[geb13779-bib-0012] Don, A. , Schumacher, J. , & Freibauer, A. (2011). Impact of tropical land‐use change on soil organic carbon stocks–a meta‐analysis. Global Change Biology, 17(4), 1658–1670.

[geb13779-bib-0013] Georgiou, K. , Jackson, R. B. , Vindušková, O. , Abramoff, R. Z. , Ahlström, A. , Feng, W. , Harden, J. W. , Pellegrini, A. F. , Polley, H. W. , & Soong, J. L. (2022). Global stocks and capacity of mineral‐associated soil organic carbon. Nature Communications, 13(1), 3797. 10.1038/s41467-022-31540-9 PMC9249731 35778395

[geb13779-bib-0014] Gerber, W. , Rickli, C. , & Graf, F. (2002). Surface erosion in cleared and uncleared mountain windthrow sites. Forest Snow and Landscape Research, 77(1–2), 109–116.

[geb13779-bib-0016] Goldstein, A. , Turner, W. R. , Spawn, S. A. , Anderson‐Teixeira, K. J. , Cook‐Patton, S. , Fargione, J. , Gibbs, H. K. , Griscom, B. , Hewson, J. H. , & Howard, J. F. (2020). Protecting irrecoverable carbon in Earth's ecosystems. Nature Climate Change, 10(4), 287–295.

[geb13779-bib-0017] Hagedorn, F. , Gavazov, K. , & Alexander, J. M. (2019). Above‐and belowground linkages shape responses of mountain vegetation to climate change. Science, 365(6458), 1119–1123. 31515385 10.1126/science.aax4737

[geb13779-bib-0018] Harris, N. L. , Gibbs, D. A. , Baccini, A. , Birdsey, R. A. , De Bruin, S. , Farina, M. , Fatoyinbo, L. , Hansen, M. C. , Herold, M. , & Houghton, R. A. (2021). Global maps of twenty‐first century forest carbon fluxes. Nature Climate Change, 11(3), 234–240.

[geb13779-bib-0019] Hedges, L. V. , Gurevitch, J. , & Curtis, P. S. (1999). The meta‐analysis of response ratios in experimental ecology. Ecology, 80(4), 1150–1156.

[geb13779-bib-0020] Hengl, T. , Mendes de Jesus, J. , Heuvelink, G. B. , Ruiperez Gonzalez, M. , Kilibarda, M. , Blagotić, A. , Shangguan, W. , Wright, M. N. , Geng, X. , & Bauer‐Marschallinger, B. (2017). SoilGrids250m: Global gridded soil information based on machine learning. PLoS One, 12(2), e0169748. 28207752 10.1371/journal.pone.0169748 PMC5313206

[geb13779-bib-0021] Holloway, J. E. , Lewkowicz, A. G. , Douglas, T. A. , Li, X. , Turetsky, M. R. , Baltzer, J. L. , & Jin, H. (2020). Impact of wildfire on permafrost landscapes: A review of recent advances and future prospects. Permafrost and Periglacial Processes, 31(3), 371–382.

[geb13779-bib-0022] Hong, S. B. , Yin, G. D. , Piao, S. L. , Dybzinski, R. , Cong, N. , Li, X. Y. , Wang, K. , Penuelas, J. , Zeng, H. , & Chen, A. P. (2020). Divergent responses of soil organic carbon to afforestation. Nature Sustainability, 3(9), 694.

[geb13779-bib-0023] Hume, A. M. , Chen, H. Y. , & Taylor, A. R. (2018). Intensive forest harvesting increases susceptibility of northern forest soils to carbon, nitrogen and phosphorus loss. Journal of Applied Ecology, 55(1), 246–255.

[geb13779-bib-0024] IPCC . (2019). IPCC 2019 Refinement to the 2006 IPCC Guidelines for National Greenhouse Gas Inventories (Vol. 4). World Meteorological Organization.

[geb13779-bib-0025] James, J. , & Harrison, R. (2016). The effect of harvest on Forest soil carbon: A meta‐analysis. Forests, 7(12), 308.

[geb13779-bib-0026] James, J. , Page‐Dumroese, D. , Busse, M. , Palik, B. , Zhang, J. W. , Eaton, B. , Slesak, R. , Tirocke, J. , & Kwon, H. (2021). Effects of forest harvesting and biomass removal on soil carbon and nitrogen: Two complementary meta‐analyses. Forest Ecology and Management, 485, 118935.

[geb13779-bib-0027] Johnson, D. W. , & Curtis, P. S. (2001). Effects of forest management on soil C and N storage: Meta analysis. Forest Ecology and Management, 140(2–3), 227–238.

[geb13779-bib-0028] Keeling, H. C. , & Phillips, O. L. (2007). The global relationship between forest productivity and biomass. Global Ecology and Biogeography, 16(5), 618–631.

[geb13779-bib-0029] Kooch, Y. , Hosseini, S. M. , Samonil, P. , & Hojjati, S. M. (2014). The effect of windthrow disturbances on biochemical and chemical soil properties in the northern mountainous forests of Iran. Catena, 116, 142–148.

[geb13779-bib-0030] Kramer, K. , Brang, P. , Bachofen, H. , Bugmann, H. , & Wohlgemuth, T. (2014). Site factors are more important than salvage logging for tree regeneration after wind disturbance in central European forests. Forest Ecology and Management, 331, 116–128.

[geb13779-bib-0031] Kramer, M. G. , Sollins, P. , & Sletten, R. S. (2004). Soil carbon dynamics across a windthrow disturbance sequence in Southeast Alaska. Ecology, 85(8), 2230–2244.

[geb13779-bib-0032] Kristensen, J. Å. , Rousk, J. , & Metcalfe, D. B. (2019). Below‐ground responses to insect herbivory in ecosystems with woody plant canopies: A meta‐analysis. Journal of Ecology, 108, 917–930.

[geb13779-bib-0033] Kurz, W. A. , Stinson, G. , Rampley, G. J. , Dymond, C. C. , & Neilson, E. T. (2008). Risk of natural disturbances makes future contribution of Canada's forests to the global carbon cycle highly uncertain. Proceedings of the National Academy of Sciences of the United States of America, 105(5), 1551–1555. 18230736 10.1073/pnas.0708133105 PMC2234182

[geb13779-bib-0034] Le Mellec, A. , Habermann, M. , & Michalzik, B. (2009). Canopy herbivory altering C to N ratios and soil input patterns of different organic matter fractions in a Scots pine forest. Plant and Soil, 325, 255–262.

[geb13779-bib-0035] Lehmann, J. , & Kleber, M. (2015). The contentious nature of soil organic matter. Nature, 528(7580), 60–68. 26595271 10.1038/nature16069

[geb13779-bib-0036] Li, J. , Pei, J. , Liu, J. , Wu, J. , Li, B. , Fang, C. , & Nie, M. (2021). Spatiotemporal variability of fire effects on soil carbon and nitrogen: A global meta‐analysis. Global Change Biology, 27(17), 4196–4206. 34101948 10.1111/gcb.15742

[geb13779-bib-0037] Lindroth, A. , Lagergren, F. , Grelle, A. , Klemedtsson, L. , Langvall, O. , Weslien, P. , & Tuulik, J. (2009). Storms can cause Europe‐wide reduction in forest carbon sink. Global Change Biology, 15(2), 346–355.

[geb13779-bib-0038] Loy, A. , & Korobova, J. (2021). Bootstrapping Clustered Data in R using lmeresampler. *arXiv preprint arXiv:2106.06568*.

[geb13779-bib-0039] Lugato, E. , Lavallee, J. M. , Haddix, M. L. , Panagos, P. , & Cotrufo, M. F. (2021). Different climate sensitivity of particulate and mineral‐associated soil organic matter. Nature Geoscience, 14(5), 295–300.

[geb13779-bib-0040] Mayer, M. , Matthews, B. , Rosinger, C. , Sandén, H. , Godbold, D. L. , & Katzensteiner, K. (2017). Tree regeneration retards decomposition in a temperate mountain soil after forest gap disturbance. Soil Biology and Biochemistry, 115, 490–498.

[geb13779-bib-0041] Mayer, M. , Prescott, C. E. , Abaker, W. E. A. , Augusto, L. , Cécillon, L. , Ferreira, G. W. D. , James, J. , Jandl, R. , Katzensteiner, K. , Laclau, J.‐P. , Laganière, J. , Nouvellon, Y. , Paré, D. , Stanturf, J. A. , Vanguelova, E. I. , & Vesterdal, L. (2020). Tamm review: Influence of forest management activities on soil organic carbon stocks: A knowledge synthesis. Forest Ecology and Management, 466, 118127.

[geb13779-bib-0042] Mayer, M. , Rusch, S. , Didion, M. , Baltensweiler, A. , Walthert, L. , Ranft, F. , Rigling, A. , Zimmermann, S. , & Hagedorn, F. (2023). Elevation dependent response of soil organic carbon stocks to forest windthrow. Science of the Total Environment, 857(Pt 3), 159694. 36302424 10.1016/j.scitotenv.2022.159694

[geb13779-bib-0043] Mayer, M. , Sandén, H. , Rewald, B. , Godbold, D. L. , & Katzensteiner, K. (2017). Increase in heterotrophic soil respiration by temperature drives decline in soil organic carbon stocks after forest windthrow in a mountainous ecosystem. Functional Ecology, 31(5), 1163–1172.

[geb13779-bib-0044] McDowell, N. G. , Allen, C. D. , Anderson‐Teixeira, K. , Aukema, B. H. , Bond‐Lamberty, B. , Chini, L. , Clark, J. S. , Dietze, M. , Grossiord, C. , Hanbury‐Brown, A. , Hurtt, G. C. , Jackson, R. B. , Johnson, D. J. , Kueppers, L. , Lichstein, J. W. , Ogle, K. , Poulter, B. , Pugh, T. A. M. , Seidl, R. , … Xu, C. (2020). Pervasive shifts in forest dynamics in a changing world. Science, 368(6494), 1–12. 10.1126/science.aaz9463 32467364

[geb13779-bib-0045] Nakagawa, S. , & Schielzeth, H. (2013). A general and simple method for obtaining *R* ^2^ from generalized linear mixed‐effects models. Methods in Ecology and Evolution, 4(2), 133–142.

[geb13779-bib-0046] Nave, L. E. , DeLyser, K. , Domke, G. M. , Holub, S. M. , Janowiak, M. K. , Kittler, B. , Ontl, T. A. , Sprague, E. , Sucre, E. B. , & Walters, B. F. (2022). Disturbance and management effects on forest soil organic carbon stocks in the Pacific northwest. Ecological Applications, 32(6), e2611. 35366042 10.1002/eap.2611

[geb13779-bib-0047] Nave, L. E. , DeLyser, K. , Domke, G. M. , Holub, S. M. , Janowiak, M. K. , Ontl, T. A. , Sprague, E. , Viau, N. R. , Walters, B. F. , & Swanston, C. W. (2022). Soil carbon in the South Atlantic United States: Land use change, forest management, and physiographic context. Forest Ecology and Management, 520, 120410.

[geb13779-bib-0048] Nave, L. E. , Vance, E. D. , Swanston, C. W. , & Curtis, P. S. (2010). Harvest impacts on soil carbon storage in temperate forests. Forest Ecology and Management, 259(5), 857–866.

[geb13779-bib-0049] Nave, L. E. , Vance, E. D. , Swanston, C. W. , & Curtis, P. S. (2011). Fire effects on temperate forest soil C and N storage. Ecological Applications, 21(4), 1189–1201. 21774423 10.1890/10-0660.1

[geb13779-bib-0050] Pan, Y. , Birdsey, R. A. , Fang, J. , Houghton, R. , Kauppi, P. E. , Kurz, W. A. , Phillips, O. L. , Shvidenko, A. , Lewis, S. L. , Canadell, J. G. , Ciais, P. , Jackson, R. B. , Pacala, S. W. , McGuire, A. D. , Piao, S. , Rautiainen, A. , Sitch, S. , & Hayes, D. (2011). A large and persistent carbon sink in the world's forests. Science, 333(6045), 988–993. 21764754 10.1126/science.1201609

[geb13779-bib-0051] Patacca, M. , Lindner, M. , Lucas‐Borja, M. E. , Cordonnier, T. , Fidej, G. , Gardiner, B. , Hauf, Y. , Jasinevičius, G. , Labonne, S. , & Linkevičius, E. (2022). Significant increase in natural disturbance impacts on European forests since 1950. Global Change Biology, 29(5), 18. 10.1111/gcb.16531 PMC10107665 36504289

[geb13779-bib-0052] Pellegrini, A. F. , & Jackson, R. B. (2020). The long and short of it: A review of the timescales of how fire affects soils using the pulse‐press framework. Advances in Ecological Research, 62, 147–171.

[geb13779-bib-0053] Phillips, H. R. P. , Guerra, C. A. , Bartz, M. L. C. , Briones, M. J. I. , Brown, G. , Crowther, T. W. , Ferlian, O. , Gongalsky, K. B. , van den Hoogen, J. , Krebs, J. , Orgiazzi, A. , Routh, D. , Schwarz, B. , Bach, E. M. , Bennett, J. M. , Brose, U. , Decaëns, T. , König‐Ries, B. , Loreau, M. , … Eisenhauer, N. (2019). Global distribution of earthworm diversity. Science, 366(6464), 480–485. 31649197 10.1126/science.aax4851 PMC7335308

[geb13779-bib-0054] Pinheiro, J. , Bates, D. , DebRoy, S. , Sarkar, D. , Heisterkamp, S. , Van Willigen, B. , & Maintainer, R. (2017). Package ‘nlme’. Linear and Nonlinear Mixed Effects Models, Version, 3(1), 274.

[geb13779-bib-0055] Pinheiro, J. C. , & Bates, D. M. (2000). Mixed‐effects models in S and S‐plus. Springer ‐ Verlag.

[geb13779-bib-0056] Prietzel, J. , Hiesch, S. , Harrington, G. , & Müller, S. (2020). Microstructural and biochemical diversity of forest soil organic surface layers revealed by density fractionation. Geoderma, 366, 114262.

[geb13779-bib-0057] Prietzel, J. , Zimmermann, L. , Schubert, A. , & Christophel, D. (2016). Organic matter losses in German Alps forest soils since the 1970s most likely caused by warming. Nature Geoscience, 9(7), 543–548.

[geb13779-bib-0058] Pugh, T. A. , Lindeskog, M. , Smith, B. , Poulter, B. , Arneth, A. , Haverd, V. , & Calle, L. (2019). Role of forest regrowth in global carbon sink dynamics. Proceedings of the National Academy of Sciences of the United States of America, 116(10), 4382–4387. 30782807 10.1073/pnas.1810512116 PMC6410874

[geb13779-bib-0059] R Core Team . (2021). R: A language and environment for statistical computing. R Foundation for Statistical Computing.

[geb13779-bib-0060] Roebroek, C. T. , Duveiller, G. , Seneviratne, S. I. , Davin, E. L. , & Cescatti, A. (2023). Releasing global forests from human management: How much more carbon could be stored? Science, 380(6646), 749–753. 37200428 10.1126/science.add5878

[geb13779-bib-0061] Šamonil, P. , Král, K. , & Hort, L. (2010). The role of tree uprooting in soil formation: A critical literature review. Geoderma, 157(3–4), 65–79.

[geb13779-bib-0062] Sayre, R. , Karagulle, D. , Frye, C. , Boucher, T. , Wolff, N. H. , Breyer, S. , Wright, D. , Martin, M. , Butler, K. , Van Graafeiland, K. , Touval, J. , Sotomayor, L. , McGowan, J. , Game, E. T. , & Possingham, H. (2020). An assessment of the representation of ecosystems in global protected areas using new maps of world climate regions and world ecosystems. Global Ecology and Conservation, 21, e00860.

[geb13779-bib-0063] Seidl, R. , Thom, D. , Kautz, M. , Martin‐Benito, D. , Peltoniemi, M. , Vacchiano, G. , Wild, J. , Ascoli, D. , Petr, M. , Honkaniemi, J. , Lexer, M. J. , Trotsiuk, V. , Mairota, P. , Svoboda, M. , Fabrika, M. , Nagel, T. A. , & Reyer, C. P. O. (2017). Forest disturbances under climate change. Nature Climate Change, 7(6), 395–402. 10.1038/nclimate3303 PMC5572641 28861124

[geb13779-bib-0064] Seidl, R. , & Turner, M. G. (2022). Post‐disturbance reorganization of forest ecosystems in a changing world. Proceedings of the National Academy of Sciences of the United States of America, 119(28), e2202190119. 35787053 10.1073/pnas.2202190119 PMC9282434

[geb13779-bib-0065] Senf, C. , & Seidl, R. (2021). Mapping the forest disturbance regimes of Europe. Nature Sustainability, 4(1), 63–U102.

[geb13779-bib-0066] Slessarev, E. W. , Mayer, A. , Kelly, C. , Georgiou, K. , Pett‐Ridge, J. , & Nuccio, E. E. (2023). Initial soil organic carbon stocks govern changes in soil carbon: Reality or artifact? Global Change Biology, 29(5), 1239–1247. 36268673 10.1111/gcb.16491 PMC10092500

[geb13779-bib-0067] Terrer, C. , Phillips, R. P. , Hungate, B. A. , Rosende, J. , Pett‐Ridge, J. , Craig, M. E. , van Groenigen, K. J. , Keenan, T. F. , Sulman, B. N. , & Stocker, B. D. (2021). A trade‐off between plant and soil carbon storage under elevated CO_2_ . Nature, 591(7851), 599–603. 33762765 10.1038/s41586-021-03306-8

[geb13779-bib-0068] Wang, J. , Taylor, A. R. , & D'Orangeville, L. (2023). Warming‐induced tree growth may help offset increasing disturbance across the Canadian boreal forest. Proceedings of the National Academy of Sciences of the United States of America, 120(2), e2212780120. 36595673 10.1073/pnas.2212780120 PMC9926259

[geb13779-bib-0069] Wang, J. A. , Baccini, A. , Farina, M. , Randerson, J. T. , & Friedl, M. A. (2021). Disturbance suppresses the aboveground carbon sink in north American boreal forests. Nature Climate Change, 11(5), 435–441.

[geb13779-bib-0070] Wu, C. , Coffield, S. R. , Goulden, M. L. , Randerson, J. T. , Trugman, A. T. , & Anderegg, W. R. (2023). Uncertainty in US forest carbon storage potential due to climate risks. Nature Geoscience, 16, 1–8.

[geb13779-bib-0071] Xu, S. , Eisenhauer, N. , Pellegrini, A. F. , Wang, J. , Certini, G. , Guerra, C. A. , & Lai, D. Y. (2022). Fire frequency and type regulate the response of soil carbon cycling and storage to fire across soil depths and ecosystems: A meta‐analysis. Science of the Total Environment, 825, 153921. 35189231 10.1016/j.scitotenv.2022.153921

[geb13779-bib-0072] Yanai, R. D. , Currie, W. S. , & Goodale, C. L. (2003). Soil carbon dynamics after Forest harvest: An ecosystem paradigm reconsidered. Ecosystems, 6(3), 197–212.

[geb13779-bib-0073] Yang, L. H. , & Gratton, C. (2014). Insects as drivers of ecosystem processes. Current Opinion in Insect Science, 2, 26–32. 32846721 10.1016/j.cois.2014.06.004

[geb13779-bib-0074] Yuwati, T. W. , Rachmanadi, D. , Turjaman, M. , Indrajaya, Y. , Nugroho, H. Y. S. H. , Qirom, M. A. , Narendra, B. H. , Winarno, B. , Lestari, S. , & Santosa, P. B. (2021). Restoration of degraded tropical peatland in Indonesia: A review. Land, 10(11), 1170.

[geb13779-bib-0075] Zhang, B. , Zhou, X. , Zhou, L. , & Ju, R. (2015). A global synthesis of below‐ground carbon responses to biotic disturbance: A meta‐analysis. Global Ecology and Biogeography, 24(2), 126–138.

[geb13779-bib-0076] Zhao, B. , Zhuang, Q. , Shurpali, N. , Köster, K. , Berninger, F. , & Pumpanen, J. (2021). North American boreal forests are a large carbon source due to wildfires from 1986 to 2016. Scientific Reports, 11(1), 7723. 33833331 10.1038/s41598-021-87343-3 PMC8032736

