# Supplementary material for: A global synthesis and conceptualization of the magnitude and duration of soil carbon losses in response to forest disturbances
Source: Glob Ecol Biogeogr. 2023 Oct 24;33(1):141–50. doi: 10.1111/geb.13779 (PMC10953364; doi:10.1111/geb.13779)
Supplement: Supplementary file 1 — Table S1. Number of studies per continent considered in the analysis. Figure S1. Explanatory power of models predicting soil organic carbon (SOC) loss from the organic layer and from mineral soil after forest disturbance. Figure S2. Soil organic carbon (SOC) losses in relation to initial SOC stock, separated by disturbance agent and soil layer. Figure S3. Forest disturbance effect on relative changes in soil organic carbon (SOC) stock depends on the initial SOC stock. [file GEB-33-141-s001.docx]

**Supplementary Information**

**A global synthesis and conceptualization of the magnitude and duration of soil carbon losses in response to forest disturbances**

The following Supplementary Information is available for this article:

**Table S1** Number of studies per continent considered in the analysis.

**Table S2** Soil organic carbon stocks in control and disturbed forest sites used in analysis.

**Fig S1** Explanatory power of models predicting soil organic carbon (SOC) loss from the organic layer and from mineral soil after forest disturbance.

**Fig S2** Soil organic carbon (SOC) losses in relation to initial SOC stock, separated by disturbance agent and soil layer.

**Fig S3** Forest disturbance effect on relative changes in soil organic carbon (SOC) stock depends on the initial SOC stock.

**Table S1** Number of studies per continent considered in the analysis.

| Continent | Studies (n) |
| --- | --- |
| Africa | 1 |
| Asia | 17 |
| Europe | 32 |
| Australia | 4 |
| North America | 93 |
| South America | 4 |

**Table S2** Soil organic carbon stocks in control and disturbed forest sites used in analysis. (see separate file)

**
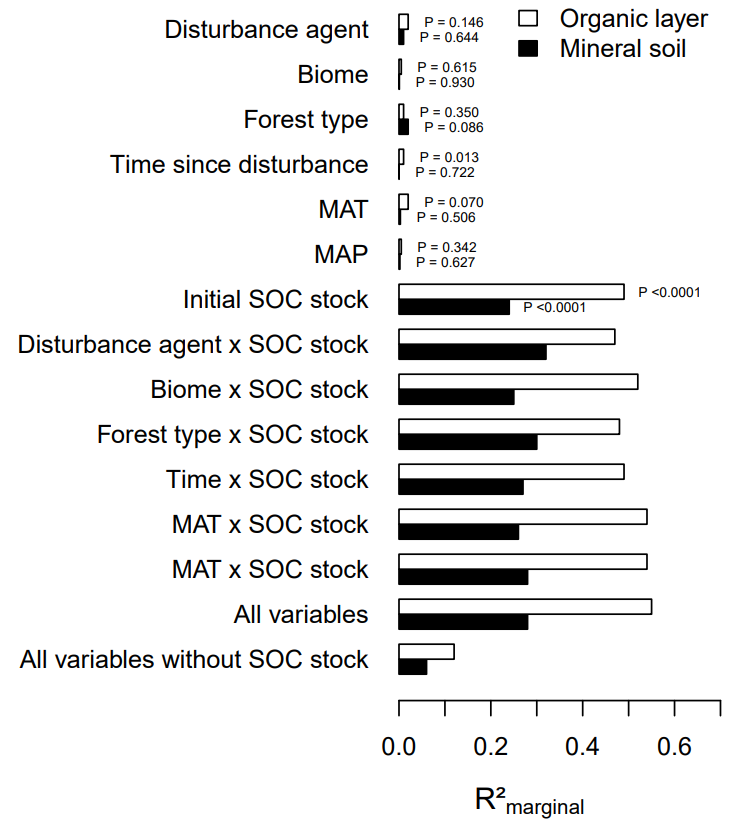
**

**Figure S1** Explanatory power of models predicting soil organic carbon (SOC) loss from the organic layer and from mineral soil after forest disturbance. Marginal R^2^ values of mixed effects models with a single predictor and with multiple predictors are displayed. For single predictors the significances (*P* values) of the model coefficients are shown. Abbreviations: MAT, mean annual temperature; MAP, mean annual precipitation.

**
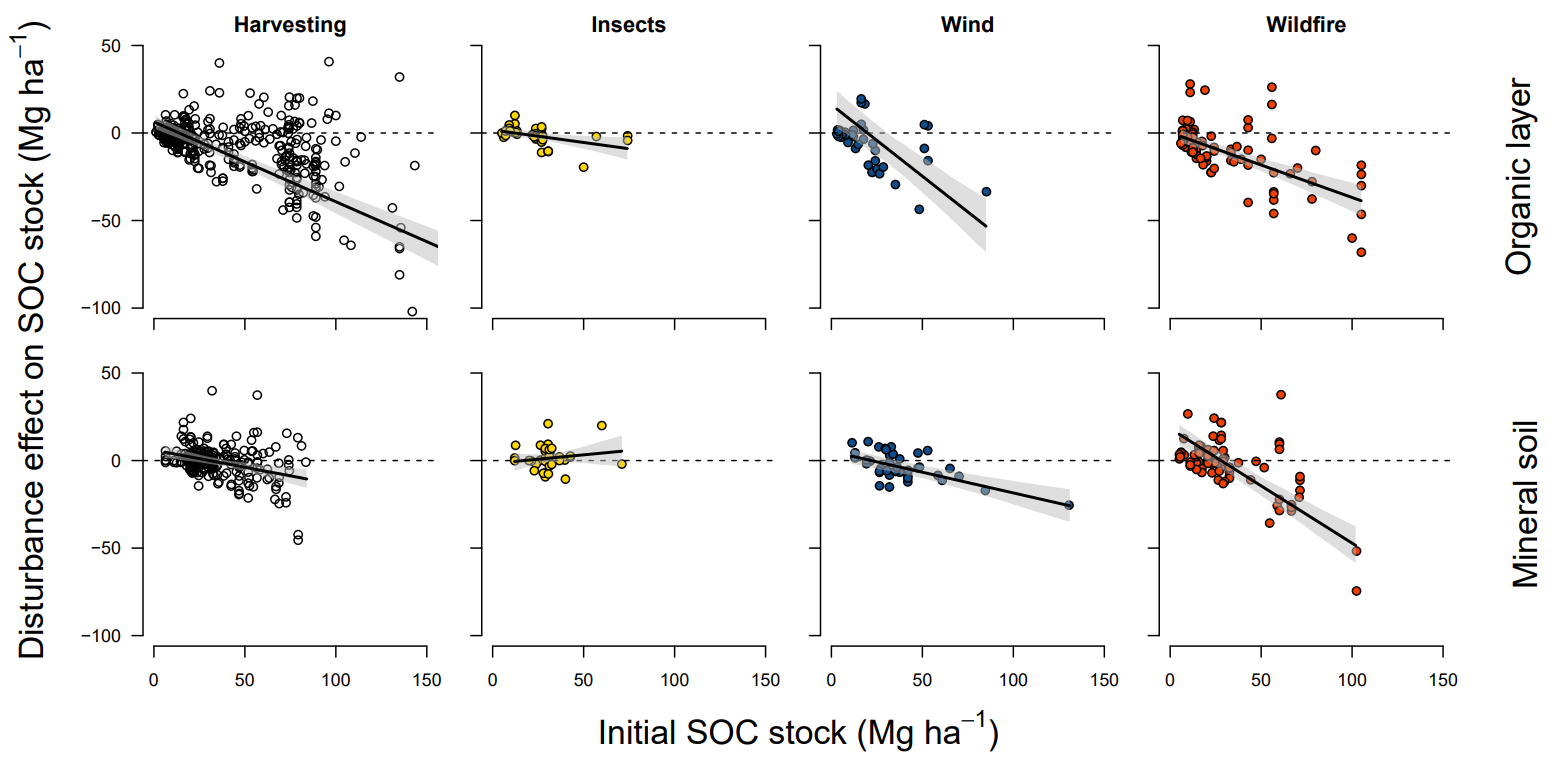
**

**Figure S2** Soil organic carbon (SOC) losses in relation to initial SOC stock, separated by disturbance agent and soil layer. The relationships were fitted using linear mixed effects models. The shaded area represents the bootstrapped 95% confidence interval.

**
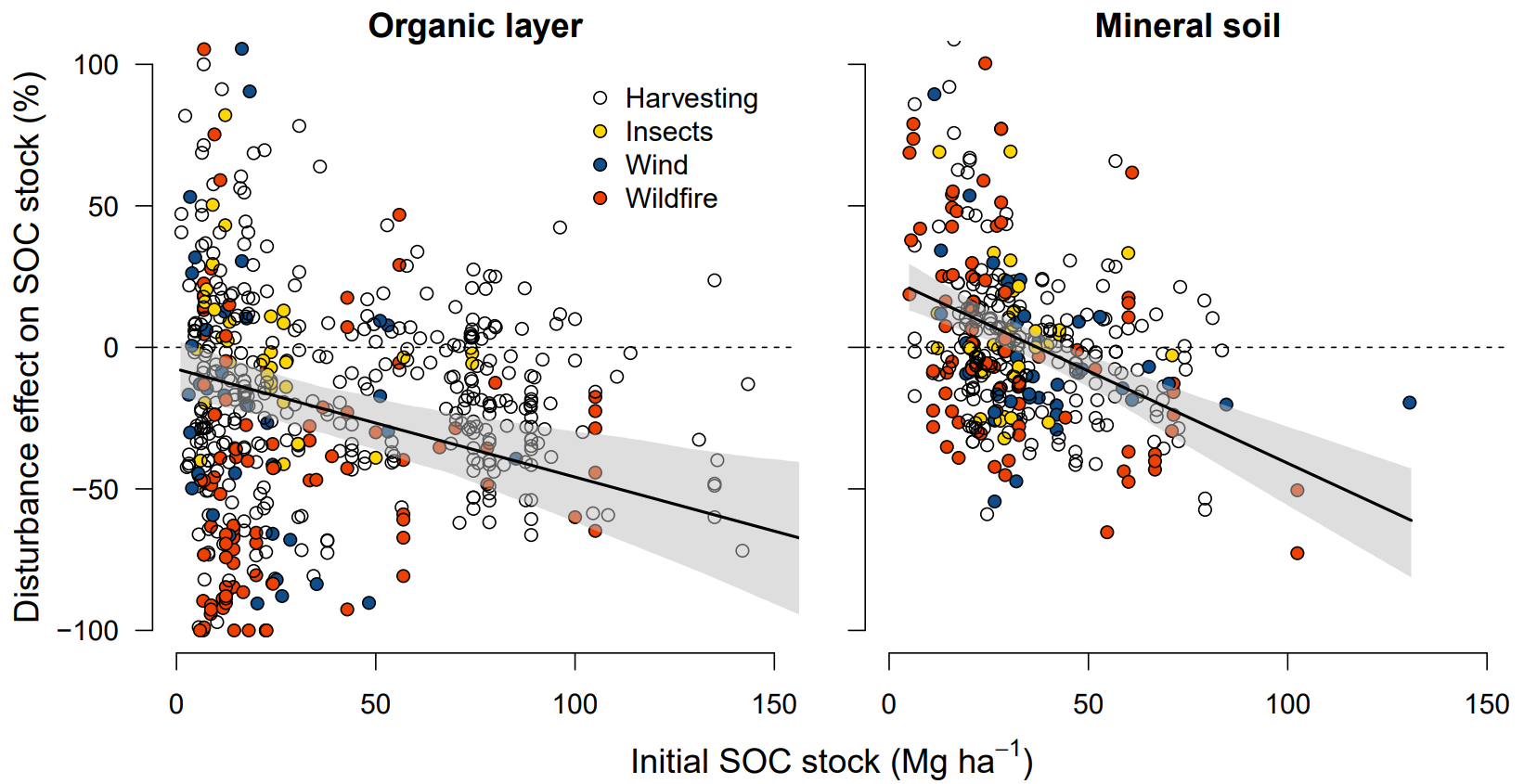
**

**Figure S3** Forest disturbance effect on relative changes in soil organic carbon (SOC) stock depends on the initial SOC stock. The relationships were fitted with linear mixed effects models (organic layer n = 542, *P* < 0.001, R² = 0.06; mineral soil n = 385, *P* < 0.001, R² = 0.12). The shaded area represents the bootstrapped 95% confidence interval. Different disturbance agents are shown in different colours.
